# Functional redundancy and stability support the resilience of the *Evernia prunastri* holobiont under urbanization

**DOI:** 10.1186/s40793-026-00886-8

**Published:** 2026-04-09

**Authors:** Panji Cahya Mawarda, Arjen Speksnijder, Daan Krijger, Juliette Berkhout, Angela Hoogenboom, Deniz Antonie Duijker, Ahmad Nuruddin Khoiri, Ken Kraaijeveld, Michael Stech, Floyd Wittink

**Affiliations:** 1 Naturalis Biodiversity Center, Darwinweg 2, 2333 CR Leiden, The Netherlands; 2 Leiden Centre for Applied Bioscience, University of Applied Sciences Leiden, Darwinweg 24, 2333 CR Leiden, The Netherlands; 3 Hortus Botanicus, Leiden University, Leiden, The Netherlands; 4 Research Center for Applied Microbiology, National Research and Innovation Agency Republic of Indonesia (BRIN), KST Soekarno, Bogor, Indonesia; 5 Bioinformatics and Systems Biology Program, School of Bioresources and Technology, King Mongkut’s University of Technology Thonburi, Bangkok, Thailand; 6 Leiden University, Leiden, The Netherlands

**Keywords:** Lichen holobiont, Lichen microbiome, Functional redundancy, Symbiosis, Urban ecology, Biosynthetic gene clusters, Lichen metagenome, Lichen symbiosis

## Abstract

**Background:**

Lichens are now recognized as holobionts comprising a mycobiont, photobiont, and diverse microbiomes, yet the functional roles of these additional microbial partners remain poorly characterized, especially under urbanization. Here, we used the epiphytic lichen *Evernia prunastri* from urban and natural areas to test the hypothesis that its resilience to urbanization is underpinned by functional stability and redundancy within its multi-kingdom consortium.

**Results:**

Using an integrated approach of amplicon and shotgun metagenomic sequencing, we found that the bacterial community structure and the functional potential of the mycobiont, bacteria, and fungi remained stable despite urbanization, highlighting stability and resistance to urban environmental stress. Furthermore, by focusing on symbiosis-related functions, we found that each partner shows tendencies toward certain roles, yet we discovered broad functional overlap, suggesting microbial contributions that buffer the symbiosis. Finally, we found that *E. prunastri* and its microbiome harbors diverse biosynthetic gene clusters with predicted ecological functions relevant for the symbiosis, spanning photoprotection, oxidative stress mitigation, nutrient acquisition, defense, and chemical communication.

**Conclusions:**

Our study provides unprecedented genomic evidence that lichen resilience is an emergent property of the integrated holobiont, where functional complementarity and redundancy among diverse symbiotic partners maintain stability under urban environmental conditions.

**Supplementary Information:**

The online version contains supplementary material available at 10.1186/s40793-026-00886-8.

## Introduction

Lichens were not recognized as dual-organism symbioses until the late 1800s, when Schwendener’s (1869) observations first revealed that each lichen thallus is formed by an association between a mycobiont (fungus) and a photobiont (green alga or cyanobacterium; sometimes both) [1]. Lichens were classified almost entirely on thallus morphology, photobiont traits, and fungal phenotypes, with nomenclature later formalized to anchor the name of each lichen to its mycobiont [2]. While earlier studies had a long-standing focus on this binary partnership, more recent studies involving microscopic, culture-based, and DNA sequencing approaches have revealed that lichen thalli also host diverse bacterial and fungal communities that are structurally integrated and stably associated with the lichen symbiosis [3–6]. This accumulation of evidence has led to a modern view of lichens as holobionts, which involve complex symbiotic consortia whose biology extends far beyond the simple partnership of a single fungus and its photobionts [7, 8]. Yet despite this conceptual shift, the functional contributions of these additional partners remain far less understood than their taxonomic diversity.

Previous studies, including ours, have shown that lichen-associated bacterial and fungal communities are diverse and host-specific [9–11], further suggesting that the lichen microbiome should be considered a fundamental and integrated component of the symbiosis. In fact, metagenomic analyses indicate that the bacterial microbiome may contribute to auxin and vitamin production, nitrogen fixation, and stress protection in lichen [12, 13]. Yet, the functional roles of the myriad microbial partners within lichens, particularly their biosynthetic capacities, remain poorly characterized for several reasons. First, only a small fraction of lichen symbioses has been examined for their bacterial communities, with intensive work concentrated in just a few model taxa such as *Lobaria pulmonaria* [6]. Only recently have broader, global surveys begun to emerge, including metagenomic analyses of hundreds of lichen holobionts sampled worldwide, e.g. [14, 15].,, which reveal extensive biosynthetic and taxonomic diversity across diverse lichen taxa and environments. Second, the recalcitrance of lichens to laboratory experimentation, including extremely slow growth and difficulties to genetically manipulate symbionts, has prevented direct functional examination [16]. Third, this problem is compounded by a research bias that has largely prioritized the bacterial component, leaving the functional roles of the equally diverse fungal communities almost entirely unexplored [17]. Together, these constraints limit our ability to predict how multi-kingdom interactions contribute to lichen symbiosis and fitness under environmental stress, an issue that becomes particularly salient under anthropogenic stressors such as urbanization.

Indeed, while many lichens can withstand harsh environmental conditions (e.g. due to desiccation tolerance), they are also among the most sensitive organisms to environmental stress, due to their lack of protective tissues and their capacity to directly absorb moisture, minerals, and gases from the atmosphere [18]. Their physiology and community composition are known to vary significantly in response to anthropogenic factors, including air quality, climate fluctuations, and pollutant deposition [19]. This sensitivity makes them valuable bioindicators in urban ecosystems. For example, physiological and morphological disturbance in lichens, including chlorophyll degradation and membrane damage, can be measured to evaluate stress associated with urbanization and elevated UV exposure [20, 21]. Additionally, in polluted urban and industrial regions, susceptible lichen species disappear, while resilient ones persist, providing a clear visual map of environmental health [22]. For years, the epiphytic lichen *Evernia prunastri* has been used as a model organism to monitor air quality and to study bioaccumulation of heavy metals and chemical pollutants in urban areas [23–26]. However, despite the lichen resilience to these pressures and urbanization, these studies rarely address the adaptation mechanism, especially at the holobiont level. This raises a key question: what stabilizes lichen multispecies symbioses under anthropogenic stress.

In this study, we address how environmental stress imposed by urbanization affects multi-kingdom contributions to *E. prunastri* function. Urbanization simultaneously alters air chemistry, temperature, moisture availability, and many other anthropogenic-related stressors. If microbial partners contribute to holobiont resilience, then urban–natural contrasts within a shared regional climatic context offer a useful framework for revealing how multi-kingdom interactions maintain function under urban-associated environmental stress. Although *E. prunastri* is sensitive to atmospheric pollutants and urban heat, its persistence in many urban environments suggests that the holobiont may buffer or resist aspects of urban stress. Since *E. prunastri* persists in urban areas, we hypothesized that (i) lichen-associated bacterial communities would be similar across urban and natural sites, and (ii) the functional profiles of the mycobiont and its associated bacterial and fungal partners would be similar across urban and natural sites. Additionally, (iii) despite occupying distinct ecological niches within the symbiosis, the mycobiont, associated fungi, and bacteria would exhibit broadly overlapping symbiotic functions, supporting the view of lichens as integrated microbial ecosystems. We further hypothesized that urban lichens would (iv) possess a diverse repertoire of biosynthetic gene clusters encoding bioactive compounds essential for stress tolerance and symbiotic stability. To test this, we applied whole-genome and metagenomic sequencing targeting the mycobiont and its associated bacteria and fungi, and amplicon sequencing targeting the lichen’s bacterial community structure, to the lichen *E. prunastri* from urban and natural areas. By integrating these data, we provide the first synthesis linking multi-kingdom functional capacities, secondary metabolism, and urban-associated environmental stress in a lichen holobiont.

## Materials and methods

### Sample collection and total lichen DNA extraction

Thalli of *E. prunastri* were collected from tree bark in an urban area in Leiden, the Netherlands, and from a nearby natural area in Meijendel, the Netherlands (Table S1). For 16S rRNA gene amplicon sequencing, 40 lichen thalli were collected in total, comprising 20 samples from each site. For metagenomic analyses, a subset of eight thalli was selected, consisting of four from the urban site and four from the natural site. Thalli selected for metagenomic sequencing were randomly chosen while ensuring representation of the natural color variation observed within each site, to avoid bias toward a specific physiological state or microhabitat condition. Prior to DNA extraction, visible debris including bark fragments, soil particles, and other macroscopic contaminants were removed manually using sterile forceps. Forceps were sterilized with 70% ethanol and flame between samples to prevent cross-contamination. No chemical surface sterilization was applied in order to preserve the native lichen-associated microbial communities. Cleaned thalli were transferred to sterile 50 mL tubes and stored at − 20 °C until further processing.

Total DNA was extracted from lichen thalli using the Quick-DNA Fecal/Soil Microbe Miniprep Kit (Zymo Research, Freiburg, Germany) according to the manufacturer’s instructions. The protocol was modified by applying three rounds of bead-beating at 5500 rpm for 45 s to enhance cell disruption, and DNA eluates were passed through Zymo-Spin III-HRC columns to reduce potential inhibitors. DNA yield was quantified with the Qubit dsDNA High Sensitivity kit (Life Technologies, Gaithersburg, MD, USA).

Extraction blank controls and PCR negative controls were included and processed alongside all samples. Negative controls were sequenced and subjected to the same bioinformatic pipeline as biological samples. No ASVs were detected in the negative controls after quality filtering, and therefore no additional contaminant filtering was required.

### Near full-length *16 S rRNA gene* sequencing

Bacterial communities were targeted by amplifying the near full-length of *16 S rRNA gene* using primers 16–27 F (5′-AGRGTTYGATYMTGGCTCAG-3′) and 16 S-1492R (5′-ACCTTGTTACGACTT-3′). PCR products were purified with the NucleoMag NGS Clean-up and Size Selection Kit (Macherey-Nagel, Düren, Germany), and fragment sizes were verified on a QIAxcel capillary electrophoresis system (Qiagen, Hilden, Germany). Concentrations of the purified fragments were determined with the Qubit dsDNA HS kit and diluted to 400 fmol (~ 260 ng for ~ 2 kb fragments). Sequencing libraries were created with the SQK-NBD114.96 kit by following the Native Barcoding Kit 96 protocol. Libraries were normalized to 10–20 fmol in 12 µL before being loaded onto R10.4.1 MinION flow cells. Sequencing was carried out on a GridION sequencer for 72 h. Basecalling, demultiplexing, and trimming of adapters and barcodes were performed with Dorado basecall server v7.6.4 using the Super-accurate basecalling v4.3.0 model, producing fastq files from the raw pod5 output.

The length and quality distribution of bacterial 16 S rRNA reads were visualized using NanoPlot and filtered with NanoFilt v.2.8.0 [27] to discard reads with a quality score below Q10 following super-accurate basecalling. Given the use of near full-length (~ 1.5 kb) reads and k-mer–based taxonomic classification (Kraken2/Bracken), this threshold was considered sufficient to retain phylogenetically informative reads while minimizing excessive read loss. The primer sequences from the filtered reads were removed using DADA2 v.1.34.0 [28]. Sequences outside the 1.4–1.6 kb range for 16 S rRNA were removed using the PRINSEQ sequence trimmer [29]. The taxonomic analyses were performed using Kraken2 and Bracken [30] against the NCBI targeted loci database for bacteria (16 S rRNA sequences). The feature table and taxonomic table, together with metadata as factors were exported using kraken-biom [31].

While this study focused on bacterial community profiling using 16 S rRNA gene sequencing, future studies incorporating fungal marker sequencing (e.g., ITS) would provide complementary insights into fungal community structure within the lichen holobiont.

### Bacterial community analyses

The bacterial sequence data from *E. prunastri* samples were rarefied to the depth of 10,504 sequences per sample. Taxa identified as archaea, chloroplasts, mitochondria, and unassigned taxa were removed resulting in 3201 amplicon sequence variants (ASVs). The remaining reads were used to calculate alpha diversity metrics, including ASV richness, Pielou’s evenness, Shannon and Simpson diversity index. Differences in bacterial community structure (beta diversity) were determined based on Bray-Curtis dissimilarity and visualized via Principal Coordinates Analysis (PCoA). Core and site-specific microbiome were identified by examining the presence and total count of shared and unique ASVs across all samples on each site. These analyses were carried out in R v.4.3.2 using the Vegan [32] and Phyloseq [33] packages.

Differences in alpha diversity parameters across the sampling sites were analyzed using Mann–Whitney U test for non-parametric data, and T-tests for parametric data. The classification of data as parametric or non-parametric was determined using the Shapiro–Wilk test and Bartlett’s test, which evaluate data normality and homogeneity of variance, respectively. Differences in community beta diversity were analyzed using the Adonis permutation test with 10,000 permutations. Additionally, we assessed the differential abundance of the bacterial taxa across sampling sites using the linear discriminant analysis effect size (LEfSe) approach, implemented with LEfSe version 1.0.8 [34].

### Shotgun metagenomic sequencing

For the shotgun metagenomic sequencing, sequencing libraries were prepared following the Nanopore Ligation Sequencing Genomic DNA protocol with the Native Barcoding Kit 24 V14 (SQK-NBD114.24). Concentrations were determined with the Qubit dsDNA HS kit and diluted to 400 ng. Final adapter ligation was performed with the SQK-NBD114.24 kit. Libraries were normalized to 300 ng in 32 µL before being loaded onto PromethION 10.4.1 flowcell placed in a PromethION 2 Solo, run on a workstation with two: NVIDIA GeForce RTXtm 4090, 128GiB memory and an AMD Ryzen Threadrippertm 7960Xs x 48. Raw pod5 data were basecalled, demultiplexed, and trimmed of barcodes and adapters using Dorado basecall server v7.6.8 with the Super-accurate basecalling v4.3.0 model. To enrich lichen-associated bacterial and fungal community reads, adaptive sequencing was performed on DNA extracted from the same *E. prunastri* thalli, using sequencing libraries as described above.

To enrich the non-host reads, adaptive sequencing was performed in MinKNOW (v.25.03.9). In MinKNOW, adaptive sampling was enabled and set to the ‘Deplete’ option, using an *E. prunastri* genome assembled from barcode01 as the depletion target (Table S1). The sequencing runs were configured to run for 72 h on a flow cell (chemistry 10.4.1). Depletion efficiency was assessed by comparing the proportion of host-mapped reads before and after adaptive sequencing. The respective proportions of host-mapped reads to total reads before and after adaptive sequencing were 59.7% and 18.1%, confirming the effective enrichment of non-host DNA.

### Long read assembly and binning

The whole genome of *E. prunastri* and metagenomic reads were subjected to quality checking to discard reads with a quality score below Q10 using nanopack v.1.1.1 [27]. To subset high-quality reads belonging to the host mycobiont *E. prunastri*, hocort v.1.2.2 [35] was applied to subsequently map those reads with the publicly available *E. prunastri* genome (GenBank accession number: GCA_003184365.1_ASM318436v1). Both mycobiont and non-mycobiont reads were assembled using Flye v.2.9.6 [36] in the -nano-hq format. Specifically for non-mycobiont sequences, the -meta parameter was applied for metagenomic assembly. Metagenomic contigs were classified as bacterial or eukaryotic using Tiara v1.0.3 [37], enabling the downstream analysis of different lichen symbionts (algal photobiont, associated bacteria, and fungi). For bacterial contigs, binning was performed with three independent algorithms (MetaBAT2, SemiBin2, and MaxBin2), using multi-sample contig coverage estimates from Fairy v0.5.8 [38]. DAS Tool [39] was then applied to integrate the results and select the highest-quality non-redundant bins. Eukaryotic contigs were binned using MetaBAT2 only, since SemiBin2 and MaxBin2 are binning tools designed for prokaryotic sequences. To assess the quality of eukaryotic bins, completeness and contamination were estimated with EukCC. Both DAS tool and EukCC were set to retain only bins with ≥ 50% completeness and ≤ 10% contamination, while lower-quality bins were treated as unbinned contigs.

### Taxonomic assignment, functional profiling, and biosynthetic gene clusters (BGCs) mining

Taxonomic classification was performed using BAT for MAGs and CAT for unbinned contigs [40]. Only sequences classified to at least the kingdom level for Bacteria and Fungi (lichen microbiome) were retained for further analysis. Sequences belonging to the phylum Chlorophyta (lichen photobiont) were not analyzed in this study, as the number of reads was not sufficient for comprehensive analysis. The CDSs were predicted using prodigal [41] for lichen bacterial unbinned contigs and lichen bacterial MAGs, and FunAnnotate [42] for mycobiont *E. prunastri* contigs, lichen fungal unbinned contigs, and lichen fungal MAGs. Functional annotation was performed using the KEGG database via GhostKOALA with DIAMOND v2.1.9 [43]. Gene-level read counts were obtained by mapping long reads to sample-specific assemblies using minimap2 (-ax lr: hq) and quantifying reads overlapping predicted coding sequences (CDS) with featureCounts. Gene prediction was performed using Prodigal for bacterial assemblies and FunAnnotate for fungal and host assemblies. Gene abundances were normalized as transcripts per million (TPM), calculated as length-normalized counts (counts divided by gene length) scaled by the sum of all length-normalized counts per sample ×10⁶. TPM values were computed in R using a custom script. The alpha and beta diversity of these genes were analyzed in the same way as the bacterial community analyses as described above. The differential abundance of the functional KOs across sampling sites was analyzed using ALDEX2 using Welch’s test [44]. To disentangle the contribution of each symbiotic partner to the lichen symbiotic system, we subset KEGG orthologs classified as iron/siderophore metabolism, defense and resistance, sulfur cycling, nitrogen cycling, phosphorous metabolism, nutrient transport, pigments and melanin, plant hormone and signaling, glycan biosynthesis and EPS production, detox and oxidative stress, vitamins and cofactors, carbon and energy metabolism, core genetic processes and growth as used also by [12, 13].

The biosynthetic gene clusters (BGCs) were predicted from lichen bacterial and fungal communities, as well as from the mycobiont *E. prunastri*, using antiSMASH v7.0 [45] with default settings. BGCs exhibiting high similarity were grouped into gene cluster families (GCFs) using BiG-SLiCE [46]. To evaluate relatedness to known BGC, the cosine distance between each BGC and entries in the BiG-FAM database was calculated, with the smallest distance used to represent each BGC’s similarity to known clusters [47].

## Results

### The *Evernia prunastri*’s bacterial community profile in urban and natural areas

**Fig. 1:**
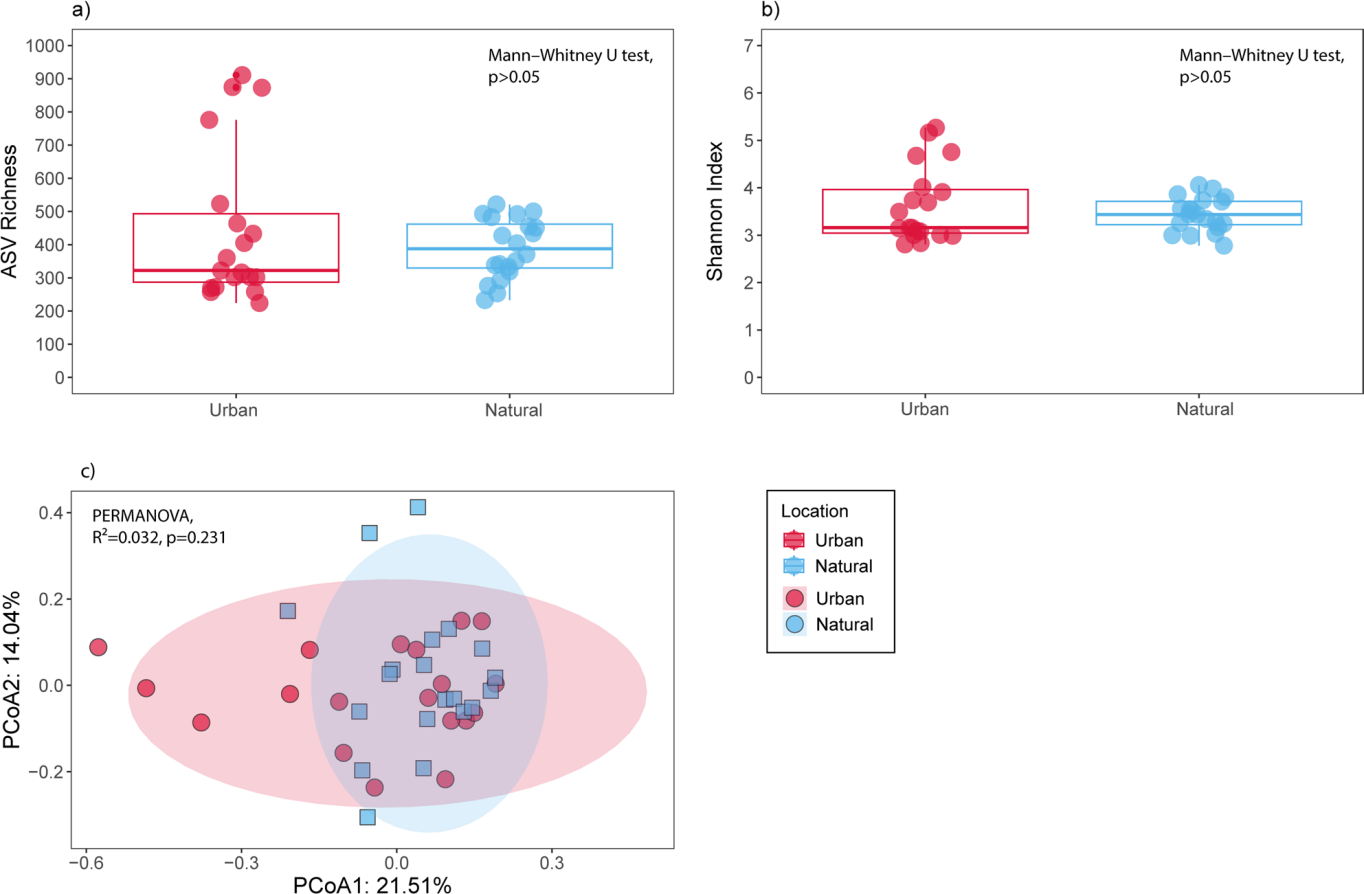
Overview of bacterial community diversity and structure in *Evernia prunastri* across urban and natural environments. Alpha diversity is represented by (**a**) ASV richness and (**b**) the Shannon diversity index for the lichen-associated bacterial communities from urban and natural sites. Bacterial community structure is depicted using a PCoA based on Bray–Curtis dissimilarities (**c**), showing the overlap between samples from the two environments. Statistical differences indicated in the panels were assessed using Mann–Whitney U tests for alpha diversity (**a**, **b**), PERMANOVA for community structure (**c**)

Alpha diversity analyses showed that the bacterial communities of *E. prunastri* are stable with respect to urbanization. ASV richness, Shannon diversity index (Fig. 1a, b, Mann–Whitney U test, *p* > 0.05), Simpson index, and Pielou’s evenness (Fig. S1a, b, Mann–Whitney U test, *p* > 0.05) did not differ between urban and natural sites, indicating the stability of the lichen bacterial diversity across environments. Similarly, beta diversity analyses revealed that the overall bacterial community structure was conserved across the urbanization gradient (Fig. 1c). Although the first two PCoA axes explained 21.51% and 14.04% of the variance, the lichen bacteria from urban and natural samples overlapped, and no significant clustering was detected (PERMANOVA, R²=0.032, *p* = 0.231). Together, these results demonstrate that the lichen-associated bacterial diversity is stable across environments and resilient to urban disturbance, maintaining a consistent structure despite differences in land use and geographic distance.

**Fig. 2:**
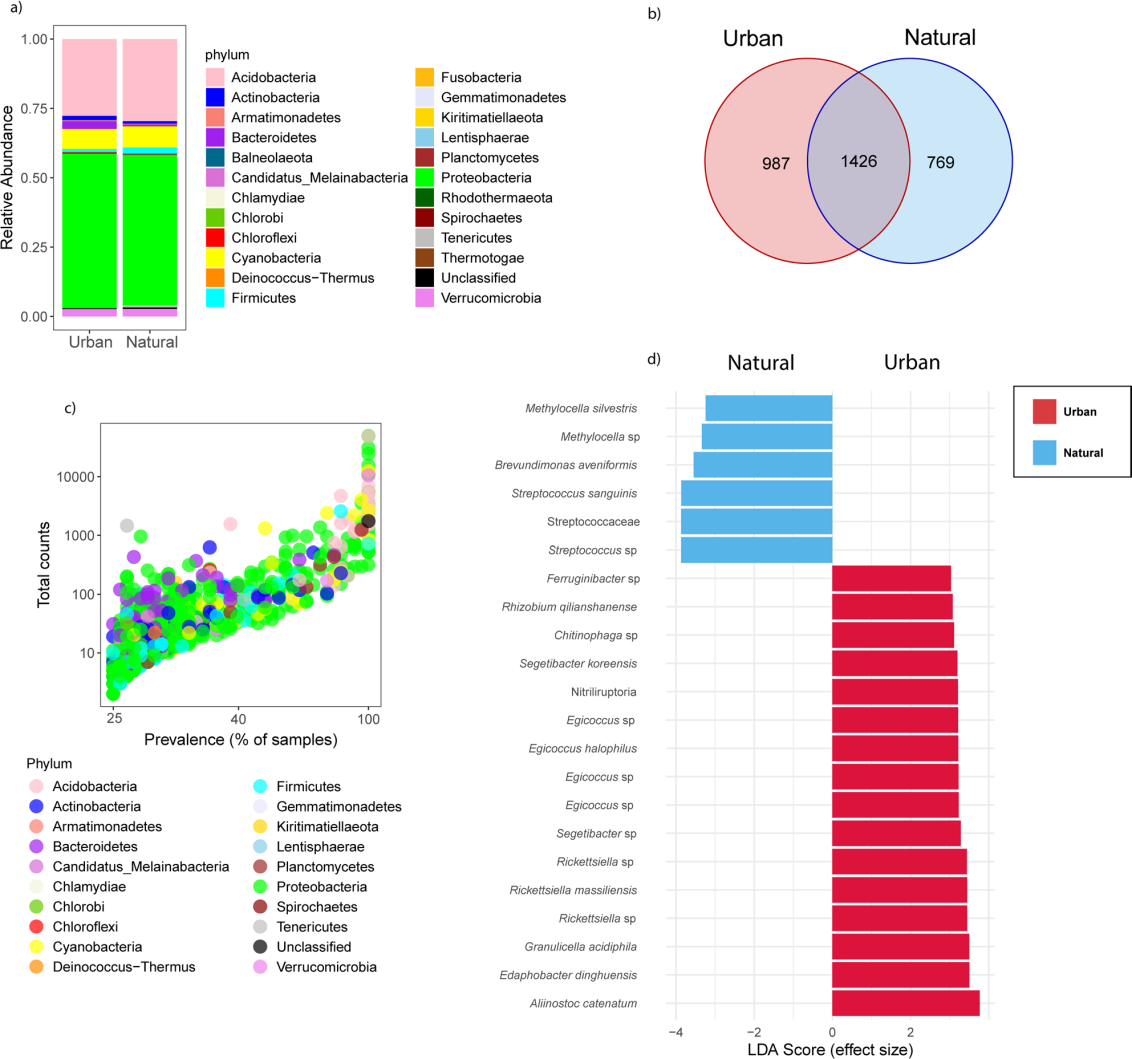
Overview of bacterial community composition and shared taxa in *Evernia prunastri* across urban and natural environments. Taxonomic composition at the phylum level is shown in (**a**) for bacterial communities associated with urban and natural lichen thalli. The distribution of shared and habitat-specific bacterial taxa is shown in (**b**), with most ASVs shared between environments and only a smaller fraction unique to each site. Patterns of prevalence and total abundance of shared ASVs are presented in (**c**), showing that shared taxa are widespread and span a broad abundance range. Differentially abundant ASVs between habitats are shown in (**d**) based on LEfSe analysis, highlighting that only a small subset of taxa differ in relative abundance between environments

To further evaluate whether the bacterial community composition of *E. prunastri* reflects microbiome stability under urbanization, we compared taxonomic composition, shared, and unique ASVs between urban and natural sites. Taxonomic classification revealed that bacterial communities of *E. prunastri* were highly conserved across urban and natural environments. At the phylum level, both habitats were dominated by Proteobacteria (~ 50% of total abundance), followed by Acidobacteria (~ 25%), Cyanobacteria (~ 10%), Verrucomicrobia (< 5%), and more than 20 additional phyla, each contributing less than 1% (Fig. 2a). Most of the community was shared between environments, with 1,426 ASVs detected in both urban and natural lichens. In addition, 987 ASVs were found exclusively in urban sites and 769 in natural sites (Fig. 2b). These unique taxa were rare, occurring in less than ~ 15% of the samples and contributing fewer than 100 counts to the overall abundance (Fig. S1c). The shared ASVs were more widespread, occurring in more than 25% of all samples, and they spanned a wide abundance range from 50 to over 10,000 counts (Fig. 2c). These patterns demonstrate that the lichen-associated bacterial communities had highly conserved shared bacterial community driving similarity across sampling locations, while rare, habitat-specific taxa contribute only marginally to compositional differences.

Differential abundance analysis using LEfSe further supported the stability of the lichen-associated bacterial microbiome. Out of 3,182 ASVs, only 22 showed significant differences in relative abundance between environments, with 16 enriched in urban lichens and 6 enriched in natural lichens (Fig. 2d). Among the taxa enriched in urban samples were the nitrogen-fixing cyanobacterium *Aliinostoc catenatum*, the acidophilic and metal-resistant bacteria *Edaphobacter dinghuensis* and *Granulicella acidiphila*, and the tick-associated bacterium *Rickettsiella massiliensis*. Natural samples showed enrichment of *Streptococcus*, the plant growth–promoting bacterium *Brevundimonas aveniformis*, and the methanotroph *Methylocella silvestris*. This underscores that variation in relative abundance is limited and that the lichen microbiome composition and abundance remain largely consistent across urban and natural sites.

### Urbanization effects on the functional potential of *Evernia prunastri* and its associated microbiomes

**Fig. 3:**
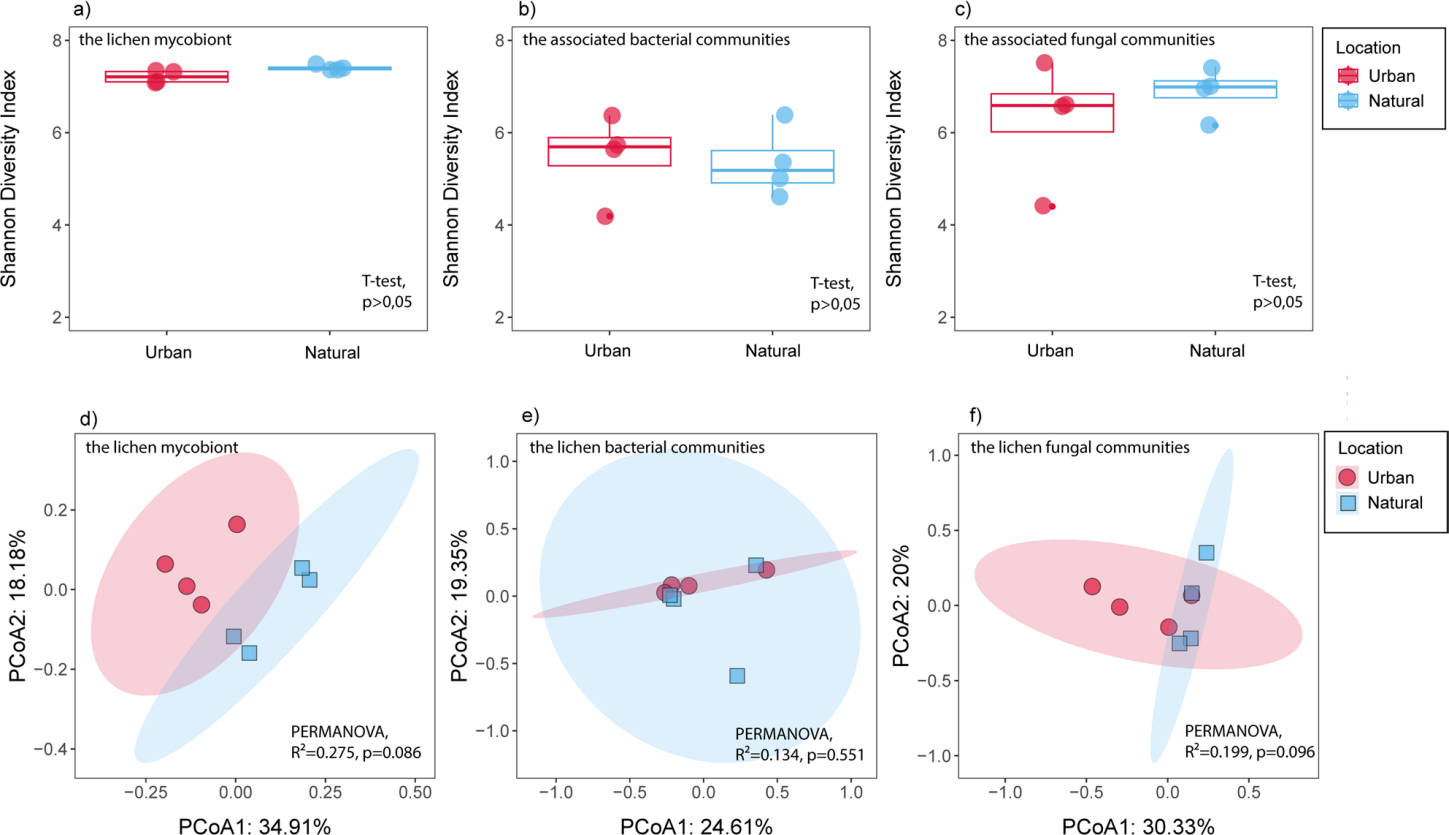
Overview of the functional diversity and functional structure of the *Evernia prunastri* holobiont across urban and natural environments. Functional alpha diversity of KEGG Orthologs (KOs) is represented by the Shannon diversity index for (**a**) the lichen mycobiont, (**b**) the associated bacterial communities, and (**c**) the associated fungal communities, all showing similar values between urban and natural samples. Functional community structure is depicted using PCoA based on Bray–Curtis dissimilarities for (**d**) the mycobiont, (**e**) the bacterial communities, and (**f**) the fungal communities. In all cases, urban and natural samples show overlapping clusters, indicating no significant functional separation between environments. Statistical differences indicated in the panels were assessed using T-tests for alpha diversity (**a–c**) and PERMANOVA for functional community structure (**d–f**).

Next, we examined the impact of urbanization on the functional potential of the lichen holobiont. For this purpose, the functional profiles of the *E. prunastri* mycobiont and its associated bacterial and fungal communities were separated to comprehensively assess the impact of urbanization on the functional potential of each symbiotic partner. The richness and Shannon diversity index of KEGG Orthologs (KOs) were similar between samples from urban and natural areas for the lichen mycobiont, as well as for its bacterial and fungal communities (Fig. 3a, b, c, *p* > 0.05). Similarly, PCoA based on Bray–Curtis dissimilarity revealed overlapping clusters of functional profiles between urban and natural areas in the lichen mycobiont (Fig. 3d; PERMANOVA, R² = 0.375, *p* = 0.086), bacterial (Fig. 3e; PERMANOVA, R²=0.032, *p* = 0.231), and fungal communities (Fig. 3f; PERMANOVA, R²=0.199, *p* = 0.096). These findings suggest that urbanization does not substantially alter the overall functional potential of the lichen holobiont.

**Fig. 4:**
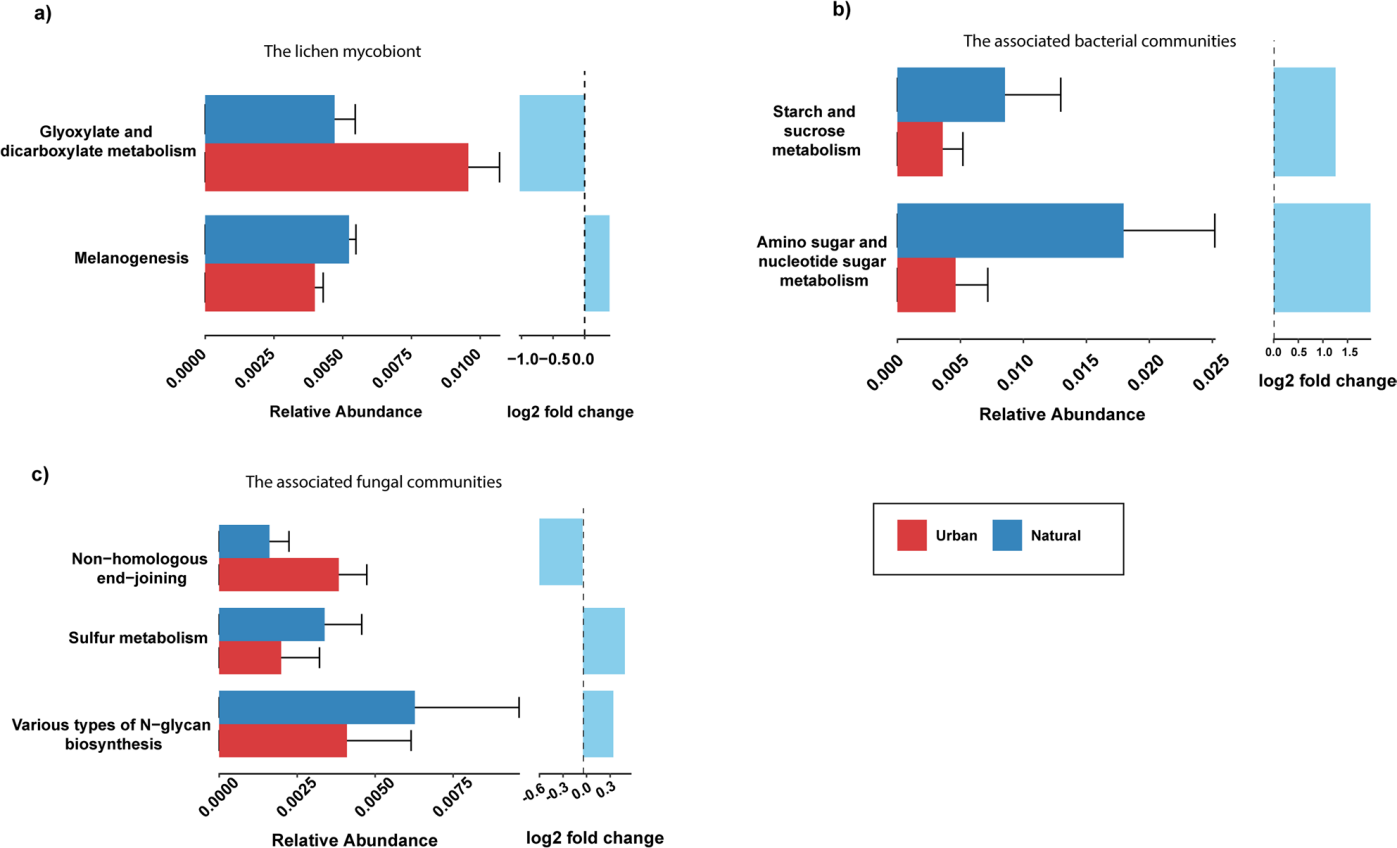
KEGG pathway-level functional differences in the *Evernia prunastri* holobiont across urban and natural environments. Differential abundance of KEGG pathways is shown for (**a**) the lichen mycobiont, (**b**) the associated bacterial communities, and (**c**) the associated fungal communities. Statistical differences indicated in the panels were assessed using ALDEx2 Welch’s t-tests (*p* < 0.05)

Furthermore, differential abundance analysis at the KEGG pathways level revealed limited functional differences between urban and natural areas. Only 2 out of 128 pathways in the *E. prunastri* mycobiont (Fig. 4a), 2 out of 231 in the bacterial communities (Fig. 4b), and 3 out of 254 in the fungal communities (Fig. 4c) showed significant differences between urban and natural samples. In the mycobiont, the glyoxylate and dicarboxylate metabolism pathway was more abundant in urban samples, whereas melanogenesis were enriched in natural samples (ALDEx2 Welch’s t-test, *p* < 0.05). In bacterial communities, starch & sucrose metabolism and amino sugar & nucleotide sugar metabolism were higher in natural than in urban lichen samples (ALDEx2 Welch’s t-test, *p* < 0.05). Finally, in fungal communities, sulfur metabolism and various types of N-glycan biosynthesis were significantly higher in natural samples, whereas non-homologous end joining DNA repair pathway (NHEJ) was higher in urban samples (ALDEx2 Welch’s t-test, *p* < 0.05). Together, these findings highlight the functional resistance of the *E. prunastri* holobiont as these changes were pathway-specific rather than systemic, suggesting that its symbiotic partners could potentially maintain functional balance despite urban environmental pressures.

### Functional potential of *Evernia prunastri* and its associated microbiomes

**Fig. 5:**
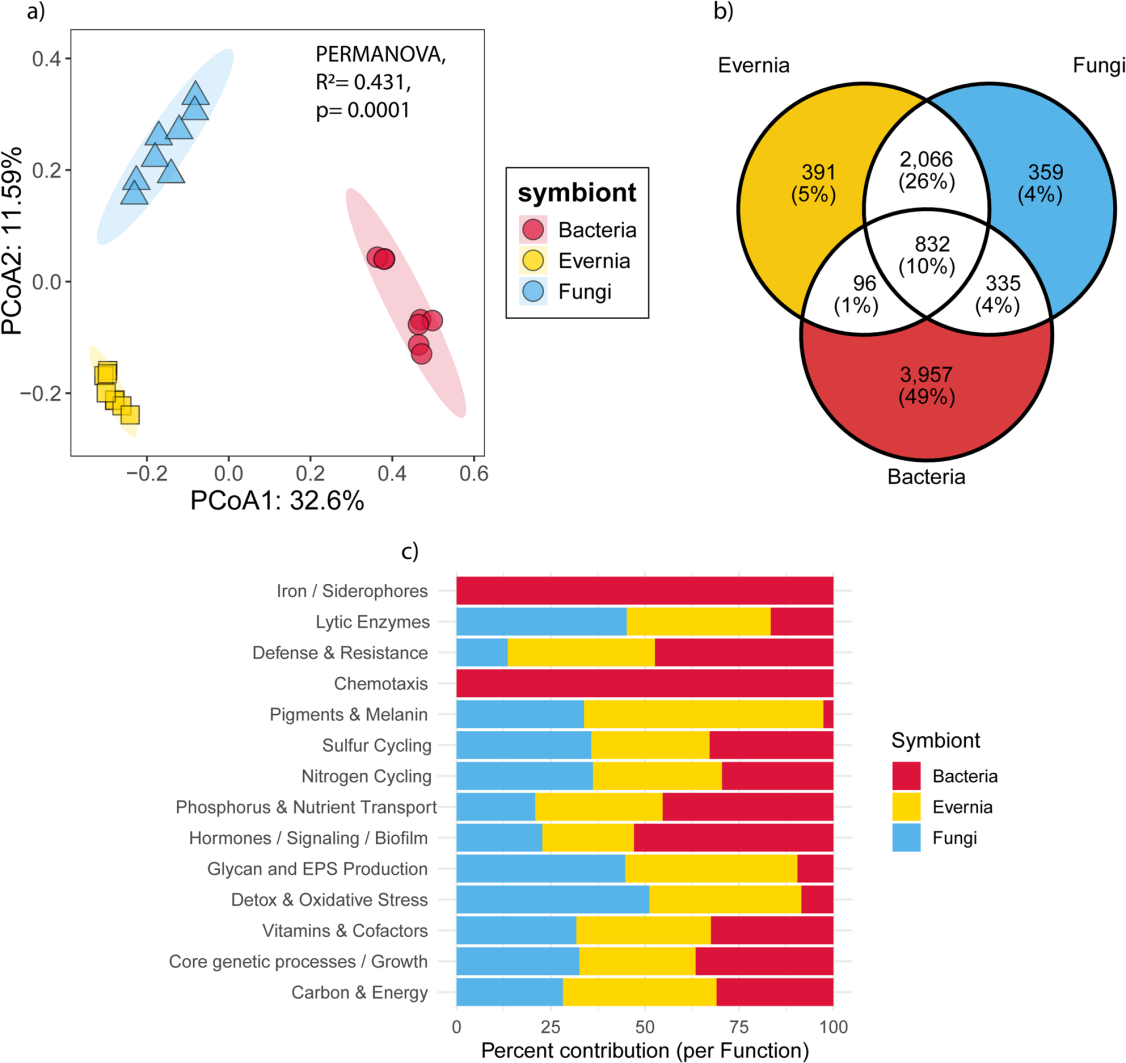
Functional differentiation and contribution of the *Evernia prunastri* holobiont. Functional profiles based on KEGG Orthologs (KOs) show clear separation among the mycobiont, associated bacteria, and associated fungi, as depicted by PCoA using Bray–Curtis dissimilarities (**a**), with significant differences confirmed by PERMANOVA (*p* = 0.0001). The total KO repertoire is shown in (**b**), illustrating the distribution of shared and unique functions across the 8036 detected KOs. Functional contributions to key symbiotic processes are presented in (**c**), highlighting tendencies to distinct roles across partners but substantial functional redundancy

The functional potential differences between the *E. prunastri* mycobiont, bacterial and fungal communities were further examined at KEGG orthologs (KO) level to disentangle the contribution of each lichen symbiotic partner to the lichen symbiotic system. The result showed significant separation of the gene functional profiles between the mycobiont, bacterial and fungal communities based on Bray-Curtis dissimilarity (Fig. 5a, PERMANOVA, R^2^= 0.431, *p* = 0.0001). Pairwise-Adonis analyses further confirmed that they differed from each other (*p* = 0.003), with the first and second axis explained 32.6% and 11.59% of the gene function variation, respectively.

A total of 8036 KOs were detected across the *E. prunastri* mycobiont and its associated bacterial and fungal communities (Fig. 5b). Of these, 832 KOs (10%) were shared among all three symbiotic components, representing the potential conserved functional core of the lichen holobiont. Indeed, most of these shared genes belong to those housekeeping genes responsible for mitochondrial biogenesis, tRNA and ribosome biogenesis, carbohydrate metabolisms, etc. (see Table S2 for the shared KOs and their associated KEGG pathways). The overlap of KOs between the mycobiont and other fungi (2,066 KOs; 26%) was greater than that between fungi and bacteria (335 KOs; 4%) or between the mycobiont and bacteria (96 KOs; 1%), suggesting stronger functional coupling between the lichenized fungus and its associated fungal partners.

The bacterial community harbored the largest number of unique KOs (3,957; 49%), potentially reflecting a more specific functional repertoire for the lichen symbiotic system (see Table S3 for the unique KOs and their associated KEGG pathways). For example, KEGG pathways for bacterial chemotaxis, biofilm formation, and beta-lactam resistance were exclusively present in this dataset. In contrast, *E. prunastri* and the associated fungi contained 391 (5%) and 359 (4%) unique KOs, respectively. We found the KEGG pathway for melanogenesis to be unique to the *E. prunastri* mycobiont, while the KEGG pathway for aflatoxin biosynthesis was solely present in fungal communities. Yet, it is important to mention that even though these KOs are unique for each symbiont, most of the KEGG pathways can still overlap, as a gene can be uniquely present in one symbiont, yet participate in the same pathway as genes from other symbionts. Detailed information on these shared and unique KOs between symbionts, including their associated KEGG pathways can be accessed in Table S2 and Table S3, respectively.

Beyond unique and shared KOs, to further examine the functional contribution of each symbiont to the lichen symbiotic system, we grouped these 8,036 KOs to KEGG pathways playing a role in lichen symbiosis based on previous studies [12, 13]. The results revealed that the symbiotic functional potential repertoire of the lichen holobiont showed distinct and essential contributions from the *E. prunastri* mycobiont, its associated fungal communities, and bacteria (Fig. 5c). Overall, the mycobiont contributed most strongly to pigment and melanin production (63.6%), consistent with its role in photoprotection and thallus pigmentation. It also played major roles in glycan and extracellular polysaccharide (EPS) biosynthesis (45.7%), highlighting its central function in maintaining structural integrity. Meanwhile, the associated fungal communities contributed most to detoxification and oxidative stress response (51.1%) and lytic enzyme production (42.6%), indicating their importance in stress mitigation and organic matter recycling. Fungi also contributed substantially to glycan and EPS biosynthesis (44.8%), complementing the mycobiont’s structural and redox functions.

Bacterial communities, in contrast, provided unique functions associated with chemotaxis (100%) and iron/siderophore acquisition (100%), reflecting specialized roles in environmental sensing and nutrient acquisition. Bacteria also contributed strongly to hormones, signaling, and biofilm formation (52.9%), as well as phosphorus and nutrient transport (45.3%), highlighting their role in facilitating interactions within the holobiont and nutrient exchange. Interestingly, bacteria also dominated defense and resistance functions (47.3%), providing protection against pathogens and environmental stressors. Despite these distinctions, functional redundancy was observed across symbionts for most of the processes. Especially, the contributions to vitamin and cofactor metabolism, core genetic processes, carbon metabolism, nitrogen cycling, and sulfur cycling were nearly equal among all partners, underscoring the cooperative nature of these fundamental metabolic functions within the holobiont. This suggests that multiple partners can perform similar functions to maintain holobiont stability. This functional redundancy likely buffers the lichen against fluctuations in environmental conditions, ensuring complementary and backup metabolic capabilities among its symbionts.

### Biosynthetic potential of *Evernia prunastri* and its associated microbiomes

**Fig. 6:**
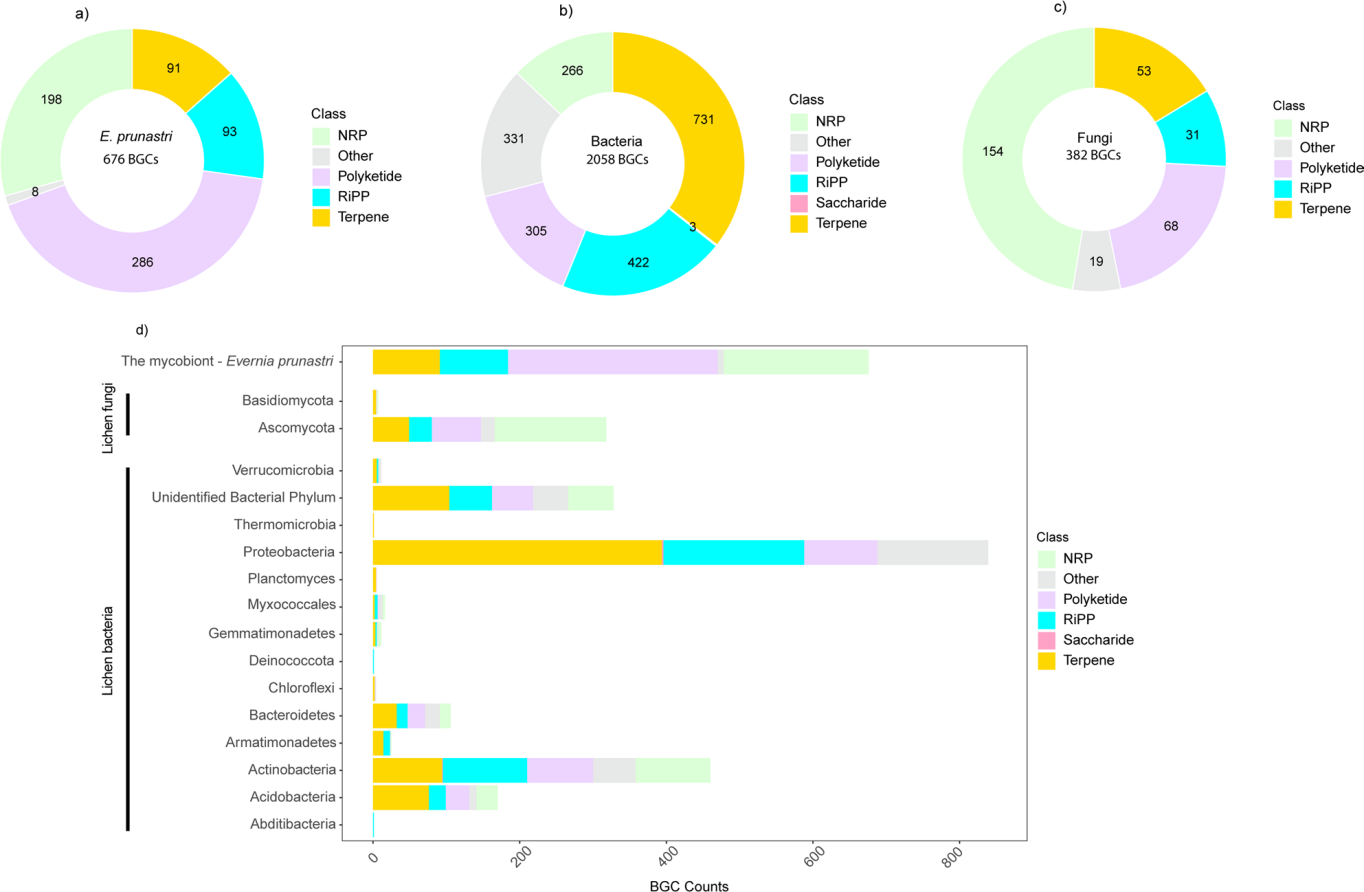
Biosynthetic gene cluster (BGC) composition across the *Evernia prunastri* holobiont. The distribution of BGC classes is shown for **a** the mycobiont *E. prunastri*, **b** associated bacterial communities, and **c** associated fungal communities. Bacteria harbor the largest number and diversity of BGCs, followed by the mycobiont and fungi. Panel **d** displays BGC counts by taxonomic group, illustrating distinct contributions of each taxon to the holobiont’s biosynthetic repertoire

The conserved bacterial communities of *E. prunastri*, together with the observed stability in functional potential across urban and natural samples, indicate that urbanization has little effect on the lichen microbiome. Consequently, we focused our analysis of biosynthetic gene clusters (BGCs) on the individual contributions of the mycobiont, associated fungi, and bacteria, rather than comparing urban versus natural sites. The result showed that the distribution of BGC classes, as assigned by antiSMASH, differed between the mycobiont and its associated microbiomes (Fig. 6). The *E. prunastri* genome contained a total of 676 BGCs, with polyketide synthase (PKS) clusters being the most abundant class (Figs. 6a, n= 268 clusters; 42.3%). Among these, type I PKS (T1PKS) was the dominant subclass, encoding predicted secondary metabolites essential for the lichen symbiotic system such as antibiotic, antioxidant, anti-inflammatory, and UV protection activities, including terreic acid, usnic acid, monascorubin, zopfielin, scytalidin, and solanapyrone A. A complete list of BGC types, their classes, subclasses, and predicted products from *E. punastri*’s genome is provided in Table S4.

Meanwhile, the lichen-associated bacteria harbored the highest number of BGCs overall (*n* = 2,058), with terpenes (*n* = 731; 35.51%) and ribosomally synthesized and post-translationally modified peptides (RiPPs; *n* = 422; 20.50%) being the most abundant classes (Fig. 6b). Proteobacteria were the dominant producers, accounting for nearly half of all bacterial BGCs (Fig. 6d, *n* = 919). This group exhibited a broad chemical repertoire, with terpenes and RiPPs as the most represented classes, followed by polyketides and nonribosomal peptides (NRPs). Some of these BGCs encoded pigments with host fitness benefits (e.g., carotenoid, zeaxanthin), antibiotics (e.g., caulonodin), pigments protecting against pathobiological damage (e.g., xanthomonadin I), and antitumor compounds (e.g., yatakemycin). Actinobacteria formed the second most biosynthetically rich group (*n* = 460; 22.35%), characterized by a more even distribution across RiPPs, NRPs, terpene, and polyketides (*n* = 91), consistent with their known role as prolific producers of bioactive metabolites. BGCs from these actinobacteria encoded iron-binding siderophores (e.g., desferrioxamine B), antibiotics (e.g., cebulantin, deoxyhangtaimycin, enduracidin, taromycin A, medermycin), antioxidant pigments (e.g., isorenieratene), anticancer compounds (e.g., granaticin), and antifungal metabolites (e.g., auroramycin). Minor contributions were also detected from Planctomycetes, Armatimonadetes, and Verrucomicrobia, each carrying mainly terpene- or RiPP-type BGCs. Collectively, these patterns highlight that while Proteobacteria are numerically dominant within the bacterial consortium, the biosynthetic potential is functionally diverse and taxonomically widespread (Fig. 6d). The full annotation of bacterial BGCs, including their classes, subclasses, and predicted products, is summarized in Table S6.

The fungal communities associated with *E. prunastri* contained a total of 382 biosynthetic gene clusters (BGCs), nearly all of which were assigned to the phylum Ascomycota, with a minor contribution from Basidiomycota (Fig. 6c). Within Ascomycota, the dominant BGC classes were NRPs and terpenes, followed by polyketides and RiPPs (Fig. 6d). The predicted secondary metabolites encoded by these clusters include the antibiotics 1,3,6,8-tetrahydroxynaphthalene, fosfonochlorin, and neosartorin; the feeding deterrent peramine, which may protect the lichen from grazing; as well as aspulvinone and naphthopyrone, compounds known for their anti-inflammatory, antioxidant, and anticancer activities. Basidiomycota members contributed only seven BGCs, primarily encoding terpenes and a few NRPs, with currently unknown secondary metabolite products (Fig. 6d). Detailed information on BGC types, classifications, and predicted secondary metabolites for fungal symbionts is available in Table S5. These findings highlight that each symbiotic partner harbors distinct BGC repertoires, with secondary metabolites that may be essential for maintaining the lichen symbiosis and hold potential for bioprospecting.

## Discussion

Urbanization affects ecosystems worldwide, imposing complex combinations of physical, chemical, and biological stressors that challenge organismal resilience [48]. Lichens, which represent long-standing models of symbiosis, offer a unique window into how multispecies associations respond to such pressures. While traditionally viewed as a dual fungus-alga partnership, lichens are now recognized as intricate holobionts that include diverse bacterial and fungal communities [6, 7]. However, whether these additional partners contribute to lichen ecological success, especially in urban environments, has not yet been extensively studied. Therefore, in this study, we comprehensively examined the structure and functional potential of *E. prunastri* and its associated microbiome across urban and natural environments to understand how urbanization influences the stability, functionality, and biosynthetic capacity of this multispecies symbiotic system.

### Bacterial community stability as a hallmark of lichen resilience to urbanization

This study aligns with our previous findings showing that urbanization has no significant impact on the diversity of lichen-associated bacterial communities [11]. Most bacterial taxa were consistently shared between sampling sites, with only a few ASVs differing in relative abundance. This compositional stability suggests that *E. prunastri* maintains a conserved core microbiome that is resilient to external perturbations. Indeed, previous studies (particularly those conducted within single ecosystems or local spatial scales) have shown that lichen-associated microbiomes tend to be host-specific despite environmental variation [9–11], reflecting a high degree of host filtering and ecological stability within the thallus microhabitat. Moreover, deterministic assembly has been reported in other lichen species, where selective pressures imposed by the host, rather than the external environment, primarily determine the microbiome composition [49, 50].

In contrast to our findings, other studies have shown that geographical location and climate can influence the structure of lichen bacterial communities, even though host specificity of lichen microbiomes was also observed [51–53]. It is important to note, however, that these studies sampled locations spanning broad altitudinal gradients, large geographical distances, and heterogeneous climatic conditions. In our study, by contrast, the spatial distance between urban and natural sites was relatively small (≤ 40 km), which may explain the limited differentiation observed. Because our sampling sites were located within the same regional climatic zone, our findings should be interpreted as reflecting urban–natural contrasts within this specific geographic context rather than representing the full heterogeneity of urban ecosystems across broader climatic or biogeographic scales. Future studies should therefore examine urbanization gradients across contrasting climatic regions, as well as across larger spatial extents, to better evaluate how environmental context modulates lichen microbiome stability.

Still, the lichen bacterial community stability suggests that the bacterial partners of *E. prunastri* are not temporary colonizers but integral components of the lichen symbiosis. Their consistent presence and stable community structure across environments indicate a long-term, co-adapted relationship with the lichen host [6, 13]. This supports a more inclusive view of the lichen as a multispecies symbiotic system rather than a dual association between a fungus and an alga [6, 7, 12]. Understanding how this stable bacterial consortium supports the host and other symbiotic partners is essential for revealing their role in lichen function and fitness under urban environmental pressures (see further discussion on complementary functional roles among symbiotic partners section).

### Functional resistance of the lichen holobiont under urban environmental pressures

In terms of function, the *E. prunastri* holobiont maintained stable functional potential across urban and natural habitats as the diversity and composition of KEGG Orthologs were similar across environments for the host, bacterial, or fungal partners. The functional stability observed in the host *E. prunastri* itself suggests the physiological versatility of lichens to adapt across environmental gradients [18, 54, 55]. Indeed, lichen-forming fungi have metabolically versatile genomes, shaped by long-term adaptation to stressors such as desiccation, UV radiation, and nutrient limitation, even though they remain highly sensitive to other environmental pressures such as air pollutants [56, 57]. Their gene content encodes the enzymatic machinery for antioxidant production, energy-adaptive metabolisms, photoprotection, and other stress-adaptive strategies which reflect resilient genomic architecture, providing the biochemical foundation to maintain their function under environmental variation [58, 59]. Subtle functional changes may instead arise from regulatory or epigenetic modulation rather than from differences in gene content [60]. This finding aligns with our study as only a limited number of pathways in the *E. prunastri* mycobiont differed significantly between urban and natural samples, despite the overall functional stability of the host. These differences appear to represent subtle metabolic adjustments rather than systemic shifts. For instance, the modest enrichment of glyoxylate and dicarboxylate metabolism in urban samples may indicate a metabolic shift toward stress-adaptive energy management. The glyoxylate cycle allows organisms to synthesize carbohydrates in a carbon-limited environment, from acetyl-CoA while bypassing CO₂-generating steps of the TCA cycle, thereby maintaining biosynthetic capacity when photosynthate input is limited [61, 62]. Urban stressors, such as air pollution, dehydration, or heat stress could reduce photobiont activity in lichens [63, 64], limiting carbon transfer to the fungal partner, triggering activation of this pathway. Although microclimatic light conditions were not measured, the enhanced melanogenesis in natural samples may indicate increased photoprotective investment, consistent with the known role of melanins in shielding hydrated thalli from UV exposure [65, 66]. Together, these adjustments represent subtle physiological tuning rather than functional disruption.

The lichen-associated bacterial and fungal communities also exhibited functional stability, retaining their metabolic potential and ecological functions under contemporary urban stress. Similar patterns of functional resistance and resilience have been reported in host-associated and environmental microbiome exposed to environmental change, highlighting the role of core microbiome and functional redundancy concept as key mechanisms [67, 68]. Our findings indeed showed the core bacterial community in *E. prunastri* was maintained by the host across sampling sites. These core microbiome members may act as the functional buffer of microbial communities by combining ecological ubiquity, high abundance, and metabolic versatility [69–71]. Their broad environmental tolerance and stable presence across habitats allow them to sustain key biochemical processes even when peripheral taxa fluctuate or are lost under stress [68, 72, 73]. Because many core taxa possess large and functionally diverse genomes, they tend to exhibit functional redundancy (where different taxa perform overlapping roles) in essential metabolic pathways such as carbon and nitrogen cycling, stress protection, and detoxification [74–76]. This redundancy and persistence confer community-level functional stability, ensuring that critical ecosystem processes are maintained under environmental perturbations [72, 77–79]. Moreover, our observation that the lichen-associated bacterial community diversity remained resistant to urbanization reinforces the established link between community resistance and functional stability in maintaining ecosystem functions [71].

While the lichen holobiont maintains overall functional stability, subtle metabolic potential differences occur among its bacterial and fungal partners as well in response to habitat context. In lichen bacterial communities for example, starch and sucrose metabolism and amino sugar and nucleotide sugar metabolism were more abundant in natural samples. Although the underlying drivers remain unclear, such shifts may reflect context-dependent differences in bacterial nutrient use or carbon processing within the thallus microenvironment, as observed in other systems [80, 81]. For lichen fungal communities, the enrichment of sulfur metabolism and various types of N-glycan biosynthesis pathways in natural samples indicates more active nutrient cycling and structural maintenance under these conditions. Sulfur metabolism supports redox homeostasis through the synthesis of cysteine, methionine, and glutathione, compounds critical for oxidative stress protection and cellular detoxification [82, 83]. Whereas, N-glycan biosynthesis affects protein folding, signaling, and cell wall integrity in fungi and lichens [84–86]. In contrast, the non-homologous end joining pathway was more abundant in urban samples, consistent with enhanced DNA repair under oxidative or chemical stress caused by pollutants and other typical urban stressors [87, 88].

### Functional roles among symbiotic partners sustain holobiont stability

While the functional potential of the *E. prunastri* holobiont remained largely conserved across urban and natural environments, our results demonstrate a clear functional diversity difference among the symbiotic partners of *E. prunastri*. This indicates that mycobiont, associated fungi, and bacteria contribute distinct metabolic capacities to the lichen symbiotic system, supporting the concept of lichen as complex microbial ecosystems [6–8, 89]. Despite these differences, a conserved set of shared genes (10% of all KOs) underpins the holobiont’s house-keeping functions, encompassing essential cellular processes such as carbohydrate metabolism, ribosome and tRNA biogenesis, and mitochondrial activity. This suggests that, while the symbiotic partners occupy different metabolic niches, they are integrated into a coordinated system that supports overall symbiotic stability [6].

Indeed, the distinct yet complementary roles of each symbiont in our study align with the previous findings showing that the mycobiont, together with its associated bacterial and fungal communities, collectively enhances the overall fitness of the lichen holobiont as an integrated symbiotic system [16, 90]. Our finding also showed that the *E. prunastri* mycobiont mainly contributes to pigment and melanin production, glycan and extracellular polymeric substance (EPS) biosynthesis, providing structural integrity, UV protection, and metabolic support for the consortium. The mycobiont provides structural support through EPS production, which (together with bacterial and fungal communities) forms a complex biofilm-like extracellular matrix that binds the symbiotic partners together [91–93]. This matrix functions both as an exoskeletal framework that stabilizes the lichen thallus and as an active interface for secondary metabolite production, cell–cell recognition, and signaling [6]. Additionally, the melanic compounds act as sunscreens, reducing UV-A, UV-B, and visible radiation, thereby protecting the photobiont layer from light-induced damage [94, 95]. These features reinforce the long-standing concept of the mycobiont as the structural and physiological “home” of the lichen symbiosis, providing the protected and metabolically active habitat in which its microbial partners coexist and interact. Although we did not examine the algal photobiont in this study, future work integrating the photosynthetic partner will be essential to establish a complete picture of the lichen holobiont’s functional architecture.

Meanwhile, this study showed that the associated fungal communities complement the function through detoxification, oxidative stress management, and organic matter recycling within the lichen. Although the ecological roles of lichenicolous and endolichenic fungi within the lichen symbiotic system remain poorly characterized, our findings provide functional evidence that these fungal associates contribute actively to this system. The detected pathways related to nutrient turnover, redox regulation, and stress protection mirror those typically observed in plant endophytic and epiphytic fungal communities, which are known to enhance host resilience and metabolic flexibility [96–98]. It is also important to note that their enrichment in lytic enzyme production provides empirical support for the concept proposed by [6], which describes lichen-associated fungal communities as “recyclers” within the lichen system. This activity may enable fungal partners to decompose environmental particulates or senescent lichen tissues, functioning as internal recyclers that sustain elemental fluxes within the thallus microecosystem.

Bacterial communities display a different ecological niche within the symbiosis, characterized by specialized functions in chemotaxis and siderophore-mediated iron acquisition, both exclusively bacterial in origin. These capabilities reflect bacterial responsiveness to microenvironmental gradients and resource scarcity, a trait often linked to colonization success and stress adaptation in the lichen holobiont [12, 13]. Their high contribution to hormone signaling, biofilm formation, and nutrient transport pathways may suggest that bacteria facilitate cross-kingdom communication and nutrient redistribution across the lichen thallus [6, 9]. The strong bacterial signal in phosphorus cycling and defense and resistance pathways further indicates that bacteria serve as both resource mediators and protective agents, scavenging limiting elements while producing antimicrobial compounds that suppress potential invaders [12, 99, 100]. In this sense, bacterial partners may act as metabolic and defensive sentinels within the lichen, maintaining nutrient flow and ecological balance.

Despite these partner-specific roles, we observed substantial overlap across symbionts for many pathways, indicating a high degree of functional redundancy. Such redundancy is a key feature of stable microbial systems, enabling multiple partners to perform similar functions and thus buffer the holobiont against environmental fluctuations [101–103]. This “multi-layered” redundancy likely ensures that critical processes (e.g., detoxification, nutrient recycling, and stress protection) remain robust even if one symbiont’s contribution is compromised. Therefore, we propose that the coexistence of complementary specialization and functional redundancy provides a dual mechanism for maintaining the lichen holobiont’s functional stability and ecological resilience under environmental variability. It is important to note, however, that holobiont-level functional resilience does not always imply tolerance to all environmental pressures; for example, historical SO₂ pollution caused major declines of sensitive epiphytic lichens such as *E. prunastri* [104, 105].

### Distinct biosynthetic repertoires reveal hidden chemical diversity and potential for bioprospecting

Our analysis revealed that the lichen holobiont harbors diverse biosynthetic potential, distributed across the mycobiont, associated fungi, and bacteria. The dominance of polyketide synthase (PKS) clusters in *E. prunastri* agrees with previous genomic and metabolomic studies showing that PKS-derived compounds, formed the predominant secondary metabolites in *E. prunastri* and other lichens [106–108]. Many of these PKS are known to accumulate as crystals in the upper cortex or medulla of the lichen thallus [109–112]. The predicted PKS compounds in our dataset such as usnic acid, terreic acid, and anthraquinones could function as photoprotectants, scavengers of free radicals, and signaling molecules within the thallus [89, 113, 114]. Previous work also showed that these metabolites help regulate the functional balance between partners, including moderating photobiont density and deterring fungal competitors, further highlighting the mycobiont’s role in maintaining the chemical and structural integrity of the lichen holobiont [115, 116].

The extensive bacterial biosynthetic repertoire, particularly among Proteobacteria and Actinobacteria, expands this secondary metabolite landscape beyond the canonical lichen compounds. These taxa are known producers of terpenes, nonribosomal peptides, and RiPPs, metabolite classes with broad ecological and pharmacological relevance [117, 118]. This aligns with large-scale analyses showing that actinomycetes account for roughly 45% of all described microbial natural products, with *Streptomyces* species as the major contributors [119, 120]. Genome-wide studies also demonstrated that Actinobacteria and multiple Proteobacterial orders, including Myxococcales and Burkholderiales, harbor some of the highest numbers of NRPS and PKS biosynthetic gene clusters among bacteria [120, 121]. Such biosynthetic potential is reflected in lichen systems as well [122]. In the previous metabolomic study, approximately 17.6% of the detected compounds in lichen thallus extracts were attributed specifically to bacteria, surpassing the proportion linked to fungi, and more than half of the detected molecules did not match products from any cultured isolates, underscoring the hidden chemical diversity within the lichen microbiome [123].

Many of the predicted bacterial metabolites identified here, such as siderophores, pigments, and antibiotics, likely support the holobiont’s nutrient acquisition, oxidative stress protection, and defense against pathogens as observed in other studies [12, 13]. Other bacterial-derived metabolites have been shown to mediate chemical communication between symbionts, influence photobiont activity, and protect the thallus from microbial invasion [6, 100]. The presence of iron-binding, signaling-related, and metabolically-relevant BGCs likely suggests that bacteria act not merely as temporary residents but as metabolically integrated partners that sustain lichen health and fitness to environmental changes [118].

Meanwhile, the fungal communities (dominated by Ascomycota) contribute to biosynthetic potential richness in non-ribosomal peptides, terpenes, and polyketides, including compounds with known antibiotic, antioxidant, and feeding deterrent activities. These patterns mirror those found in other endolichenic or plant-associated fungi, where such metabolites function in stress tolerance and defense [124–126]. Yet, lichen-associated fungal communities remain taxonomically and chemically underexplored, with only a small fraction of their predicted diversity characterized to date [127, 128]. The identification of compounds such as fosfonochlorin and peramine indicates potential ecological roles in microbial competitors [129] and deterring grazers [130] respectively, while compounds like aspulvinone and naphthopyrone may buffer oxidative stress [131] within the thallus microenvironment. Together, these results suggest that fungal symbionts enhance the lichen’s adaptive capacity by supplying bioactive molecules that complement those of the mycobiont and associated bacteria, providing new insight into the largely unexplored chemical potential of lichen fungal communities.

The collective diversity of BGCs across all symbiotic partners highlights the lichen holobiont as an underexplored source of natural products with biotechnological potential. Although lichens have long been recognized for producing unique polyketides and depsides [128, 132], our findings demonstrate that their associated microbiomes may substantially expand this repertoire. Recent advances in metagenomic mining have shown that many microbial BGCs in symbiotic systems remain cryptic under standard laboratory conditions but can be activated under environmental stress or host signaling [106]. Hence, the *E. prunastri* holobiont represents a promising system for uncovering novel bioactive compounds through integrative genome mining and metabolomic approaches.

## Conclusions

In summary, our study provides a comprehensive view of how the *E. prunastri* mycobiont and its associated microbiomes may respond to urban environmental conditions. The lichen holobiont maintained compositional and functional stability across urban and natural habitats, highlighting the lichen ecological resistance through functional redundancy and host filtering that jointly buffer the symbiosis against contemporary environmental perturbation. Furthermore, by focusing on symbiosis-related functions, we found that each partner shows tendencies toward certain roles, yet their capacities still overlap broadly, reinforcing *E. prunastri* as an integrated microbial ecosystem rather than a simple binary symbiosis. Meanwhile, the biosynthetic gene clusters reveal that *E. prunastri* harbors diverse bioactive compounds with ecological functions spanning photoprotection, oxidative stress mitigation, nutrient acquisition, competitive interactions, and chemical communication. This finding adds to growing evidence that lichen-associated microbes represent an underexamined source for secondary metabolites exploration. Looking forward, several research avenues are thus needed. First, future studies must integrate the photobiont into genomic, transcriptomic, and metabolomic analyses for resolving how photosynthetic partners affect nutrient fluxes, stress responses, and secondary metabolite dynamics within the holobiont. Second, experiments that track metabolite production under controlled environmental stressors, such as desiccation, heat pulses, or pollutant exposure, will be essential for connecting specific BGCs to ecological function. Finally, expanding urbanization gradients across distinct climatic regions will help determine the extent to which the resilience patterns observed here are generalizable beyond this regional context and clarify how environmental context influences the stability of lichen-associated microbiomes. Comparative analyses integrating publicly available multi-regional datasets, ideally harmonized through standardized sampling and sequencing frameworks, will further be essential to robustly test the broader generality of these patterns across urban and broader climatic regions.

## Supplementary Information

Supplementary Material 1.

Supplementary Material 2.

Supplementary Material 3.

Supplementary Material 4.

Supplementary Material 5.

Supplementary Material 6.

Supplementary Material 7.

## Data Availability

Raw sequences were submitted to the NCBI Sequence Read Archive (SRA) and are available under the BioProject ID PRJNA1398875. The scripts for analyses are available at https://github.com/panji-cahya/lichen-bgc-microbiome.
