# Supplementary material for: Functional redundancy and stability support the resilience of the Evernia prunastri holobiont under urbanization
Source: Environ Microbiome. 2026 Apr 9;21:65. doi: 10.1186/s40793-026-00886-8 (PMC13169696; doi:10.1186/s40793-026-00886-8)
Supplement: Supplementary file 1 — Supplementary Material 1. [file 40793_2026_886_MOESM1_ESM.docx]

## Supplementary Information

Article title: Functional redundancy and stability support the resilience of the *Evernia prunastri* symbiotic system under urbanization

Authors: Panji Cahya Mawarda^1,2,3,4^, Arjen Speksnijder^1,2^, Daan Krijger^2^, Juliette Berkhout^2^, Angela Hoogenboom^2^, Deniz Duijker^2^, Ahmad Nuruddin Khoiri^5^, Ken Kraaijeveld^2^, Michael Stech^1,6^, Floyd Wittink^2^

The following Supplementary Information is available for this article:

**Fig.S1** Overview of bacterial community diversity and structure in Evernia prunastri across urban and natural environments. Alpha diversity is represented by (a) Simpson diversity index and (b) the Pieleou’s evenness index for the lichen associated bacterial communities from urban and natural sites. Patterns of prevalence and total abundance of unique ASVs are presented in (c) for urban areas and (d) for natural areas, showing that unique bacterial taxa are low in both prevalence and abundance.


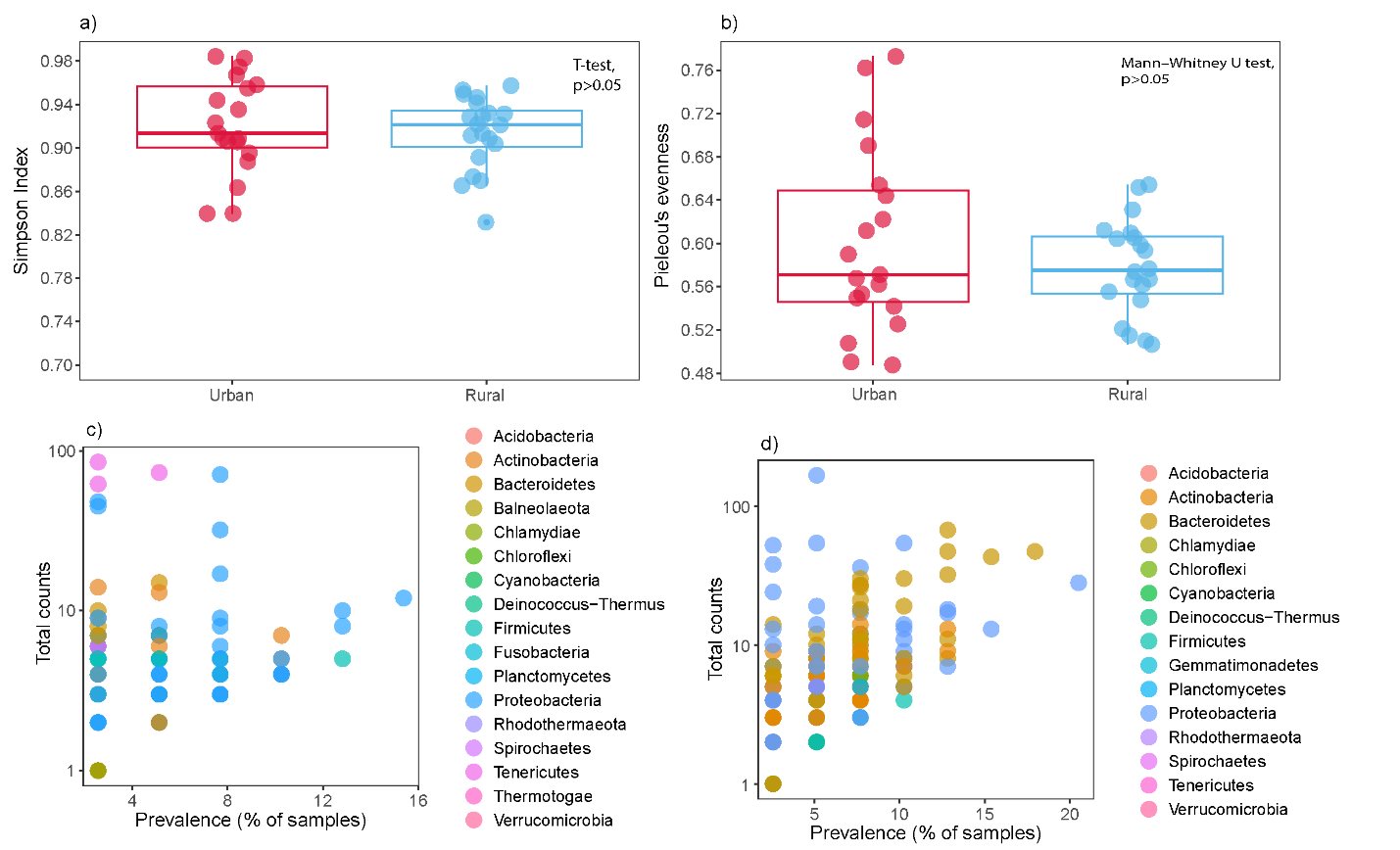


**All these tables are available as Microsoft excel files.**

**Table S1** Sample information for the lichen *Evernia prunastri* collected in urban and natural environments.

**Table S2** Shared KOs and associated KEGG pathways among the lichen symbionts .

**Table S3** Shared KOs and associated KEGG pathways in each lichen symbionts.

**Table S4** BGC types and predicted products in the mycobiont

**Table S5** BGC types and predicted products in the lichen associated fungal communities.

**Table S6** BGC types and predicted products in the lichen associated bacterial communities.
